# Coupling strategy between high-index and mid-index micro-metric waveguides for O-band applications

**DOI:** 10.1038/s41598-022-22456-x

**Published:** 2022-10-19

**Authors:** Ilias Skandalos, Thalía Domínguez Bucio, Lorenzo Mastronardi, Teerapat Rutirawut, Frederic Y. Gardes

**Affiliations:** Optoelectronics Research Centre, University of Southampton, Southampton, SO17 1BJ UK

**Keywords:** Silicon photonics, Integrated optics

## Abstract

The integration of fast and power efficient electro-absorption modulators on silicon is of utmost importance for a wide range of applications. To date, Franz-Keldysh modulators formed of bulk Ge or GeSi have been widely adopted due to the simplicity of integration required by the modulation scheme. Nevertheless, to obtain operation for a wider range of wavelengths (O to C band) a thick stack of Ge/GeSi layers forming quantum wells is required, leading to a dramatic increase in the complexity linked to sub-micron waveguide coupling. In this work, we present a proof-of-concept integration between micro-metric waveguides, through the butt-coupling of a ${1.25}\,{\upmu \hbox {m}}$ thick N-rich silicon nitride (SiN) waveguide with a ${1.25}\,{\upmu \hbox {m}}$ thick silicon waveguide for O-band operation. A numerical analysis is conducted for the design of the waveguide-to-waveguide interface, with the aim to minimize the power coupling loss and back-reflection levels. The theoretical results are compared to the measured data, demonstrating a coupling loss level of ${0.5}\,\hbox {dB}$ for TE and TM polarisation. Based on the SiN-SOI interconnection simulation strategy, the simulation results of a quantum-confined Stark effect (QCSE) stack waveguide coupled to a SiN waveguide are then presented.

## Introduction

It is expected that the global Internet Protocol (IP) traffic will reach ${4.8}\,\hbox {ZB}$ per year in 2022, almost three times the global traffic reported in 2017^1^. Data centers play a pivotal role in the management of the total information processed globally. However, bulky and power-hungry electrical intra- and inter-chip elements have historically constrained their interconnect potential^2,3^, leading the research community to develop solutions based on low cost high performance photonic integrated circuits (PICs) based on silicon^4,5^.

The basis for production of silicon photonic integrated circuits has been set due to the low cost high volume silicon manufacturing infrastructure and the sub-micron size of the generated components ^6^. Given the strong refractive index contrast in the O and C telecommunication bands, compact C-band based SOI passive devices have been efficiently implemented with SOI p-i-n junction and bulk Ge or GeSi Franz-Keldysh modulators^7^. Nevertheless, the demand for compact transceivers with lower power consumption and large modulation bandwidth in the O-band^8^, has driven interests in demonstrating efficient integration of modulators based on the quantum-confined Stark effect (QCSE)^9,10^ on silicon. QCSE type modulators are formed by barrier and well layers realised through material engineering which compose the active region for operation at specific wavelengths. Those regions are primarily formed with a high content of Ge and therefore require relatively thick buffer layers to balance the strain and lower the concentration of defects going through the wells that deteriorate the performance of the device. This results in micro-metric structures that are difficult to integrate with conventional sub-micrometric waveguides already established on a variety of SOI platforms^11,12^. It is therefore of utmost importance to introduce a complementary metal-oxide semiconductor (CMOS) compatible transition structure that will provide a flexible and reliable fabrication pathway to transition from a high index thick waveguide structure (QCSE) to a waveguide system enabling seamless optical routing as well as provide a bridging path to coupling QCSE size waveguides to sub microns waveguides.

SiGe-based multiple quantum well (MQW) QCSE modulators integrated to SOI have been the subject of a number of demonstrations. One approach for waveguide integration is the selective epitaxial growth (SEG) of MQW stacks inside pre-etched recesses and the butt-coupling interconnection to silicon waveguides. This scheme was originally demonstrated by Ren et al.^13^ where a $1.5\,\upmu \hbox {m}$ thick Ge/SiGe MQW stack was integrated to a ${310}\,\hbox {nm}$ thick SOI waveguide operating in the C-band. Recently, Shrinivasan et. al. demonstrated an improved version where a ${450}\,\hbox {nm}$ thick GeSi QCSE electro-absorption modulator is integrated to a ${220}\,\hbox {nm}$ SOI waveguide through poly-Si tapers^14,15^ showing a ${60}\,\hbox {Gb/s}$ high-speed operation at ${1321}\,\hbox {nm}$. The drawback of these schemes is linked to the selective epitaxy that poses significant challenges in obtaining sufficient thickness control of the wells during epitaxy, as well as discontinuities that may appear at the material interface of the cavities, making this fabrication process and repeatability challenging. Issues with cavity interfaces has led to high insertion loss values (${15}\,\hbox {dB}$^13^, ${16}\,\hbox {dB}$^14^), however this has been recently significantly improved (${7.6}\,\hbox {dB}$^15^).

The tunability of silicon nitride (SiN) in terms of its optical and physical properties in combination with its amorphous structure, can provide a CMOS compatible co-integration solution to the problems stated above. By modifying the N/Si ratio of the material, nitrogen-rich (N-rich, *n* < 2), stoichiometric (*n* $=$ 2) or silicon-rich (Si-rich, *n* > 2) compositions are achievable, through a low-temperature (350 °C) Plasma Enhanced Chemical Vapor Deposition (PECVD) process, demonstrating low optical propagation loss^16^. Consequently, a back end of line (BEOL) integration with a micron-thick multi-layer active medium is achievable^17^. In particular, a uniform and defect-free SiN layer can be deposited inside pre-etched cavities formed in the MQW SiGe stack grown over the entire surface of an SOI wafer, forming the basis of a butt-coupling strategy. In this particular case the epitaxy of QCSE MQW is greatly simplified, as sidewalls pinching and non uniformity issues of the MQW across the wafer are avoided. Therefore SiN films can be used effectively to form a standalone waveguiding system providing a means to efficiently couple light to thick high index waveguides such as the ones used for QCSE type devices.

Silicon nitride is also of further interest due to its mid-index identity (refractive index, *n* $\sim$ 2) and its low thermo-optic coefficient making it attractive for wavelength division (de)multiplexing (WDM)^18^, given that temperature and fabrication variations do not affect significantly the behavior of the supported optical mode. Based on the above advantages, SiN can be used as the main waveguide platform or as a bridging pathway to the SOI waveguide exploiting the evanescent coupling through adiabatic tapers showing a performance of even less than $0.1\,\hbox {dB}$/transition loss^19,20^. Consequently, each platform can be used for different applications (e.g. temperature insensitive (de)multiplexing in SiN and tight bendings in SOI), exploiting the best properties of both materials.

A monolayer (butt-coupling) type integration of thick devices with an SiN circuitry entails some complexity in terms of fabrication, which makes a proof of concept a necessary initial step. To that aim, in this work we propose a butt-coupling scheme between a micro-metric N-rich SiN passive platform and an SOI waveguide operating at $1310\,\hbox {nm}$, that mimics the SiN-to-MQW interconnection of a waveguide-integrated modulator concept in terms of the thickness and the refractive indices. The N-rich composition allows the realisation of low-loss waveguides in the O-band, while compact devices with coarse wavelength division multiplexing (CWDM) capabilities can be achieved as shown in^18^. In the following sections, we describe the methodology utilised for the SiN-to-SOI interconnection, the design of the related platforms and the characterisation output we achieved in terms of the optical coupling compared to the simulated response. Finally, we present the targeted SiN-to-MQW concept together with simulation results.

## Design and fabrication

**Figure 1:**
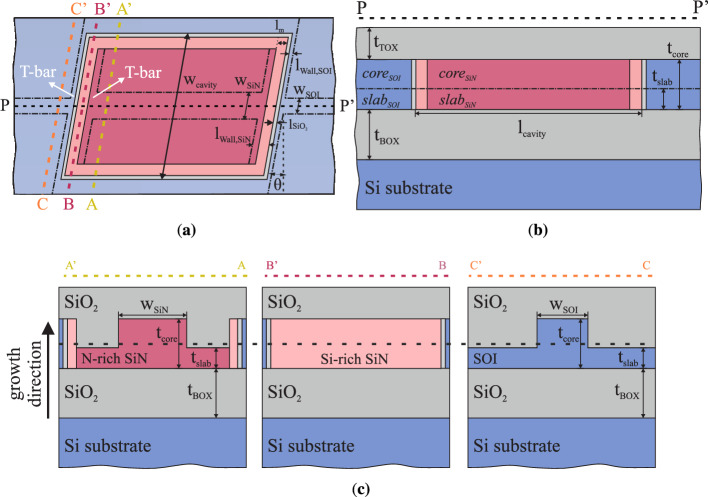
Waveguide-to-waveguide butt-coupling interconnection concept. In **(a)** the top view of the interconnection is depicted. The N-rich SiN section stands in the middle and its facets are connected to the SOI waveguides. Descriptive transverse cuts are taken to elucidate the full geometry of the device. The PP’ dashed line reveals the side view of the structure depicted in **(b)**. Cross-sections of the separate elements of the structure are disclosed in **(c)**. The cuts AA’ and CC’ reflect to the N-rich SiN and the SOI sections, respectively. BB’ refers to the Si-rich SiN layer that is set, along with the $\hbox {SiO}_{2}$ layer, between the two passive platforms towards optimal photonic coupling.

Figure 1 depicts the waveguide-to-waveguide (SiN-to-SOI) interconnection concept. Specifically, the top-view in Fig. 1a shows a straight N-rich SiN waveguide (*n* $=$ 1.9) connected from both sides to SOI waveguides (*n* $=$ 3.507) in a butt-coupling scheme along the PP’ black dashed line, which refers to the propagation direction. Thin layers of silicon dioxide ($\hbox {SiO}_{2}$) (*n* $=$ 1.4467) and Si-rich SiN (*n* $=$ 2.54) layers are located at each interfaces. The $\hbox {SiO}_{2}$ layer protects the SOI waveguides during the fabrication process, while the Si-rich SiN layer is used to mitigate the refractive index difference between the N-rich SiN and the SOI waveguides. The combination of the two layers given an adjustment of their lengths, acts as a double layer anti-reflective coating (DLARC) capable of maximizing the coupling efficiency and minimizing the back-reflection level. The side view determined by the PP’ cut is depicted in Fig. 1b. The dashed lines AA’, BB’ and CC’ cross transversely the areas of the N-rich SiN waveguide, the Si-rich SiN layer and the SOI waveguide, respectively. The related cross-sections to these lines are shown in Fig. 1c. As presented, the two passive sections consist of a rib waveguide structure encapsulated in a $\hbox {SiO}_{2}$ cladding of thicknesses $t_{BOX}$ and $t_{TOX}$ for the bottom and the top layer, respectively. The waveguides are aligned along the growth direction towards the optimal matching of the fundamental TE and TM modes based on power coupling simulations. Furthermore, the thicknesses of the core $t_{core}$ and the slab $t_{slab}$, along with the widths of the waveguides $w_{SiN}$ and $w_{SOI}$, are designed towards the enhancement of the optical coupling. In addition, a tilted interface of angle $\theta$ in degrees is investigated for the minimisation of the back-reflected power. Moreover, at the interface area and parallel to the CC’ line, non etched bars of thicknesses $l_{Wall,SiN}$ and $l_{Wall,SOI}$ are investigated on both interface sides (SiN and SOI), forming what we define as a “T-bar”. The T-bar definition is required due to the overlay accuracy of $<20\,\hbox {nm}$ for the ${248}\,\hbox {nm}$ deep ultraviolet (DUV) lithography used in this work, enabling to mitigate issues linked to lithographic overlaps and associated over-etching of the slab at the interface. Consequently, the T-bar method provides a means to control the optimisation of the coupling quality by setting an optimised value for the thicknesses of the bars.

The butt-coupling scheme between the passive waveguides was theoretically investigated through a 3D-FDTD propagation study regarding the power coupling loss and the back-reflection, exploiting the commercial platform *FDTD Solutions* of Lumerical. The study concerns the propagation of the fundamental TE and TM modes along one single interface. Initial runs about the thicknesses of the core and the slab of both waveguides were conducted, while the lengths of the $\hbox {SiO}_{2}$ and Si-rich SiN layer were identified through simulation sweeps. Furthermore, a range of angles was simulated to evaluate the efficiency of the coupling and the variation of the length of the T-bar walls was examined as well, followed by further parametric analysis of the widths of the two waveguides.

All the related geometric parameters are presented in Table 1, along with the resulting values for the coupling loss and the back-reflection for the fundamental TE and TM modes. The cases of a $0^\circ$ and a ${4^\circ }$ tilted interface are shown, however only results regarding a flat interface are presented in this work. The tilted interface was shown to induce minimal improvement, while an increase in coupling loss to a level of ${0.5}\,\hbox {dB}$ was observed. Consequently, for both polarisations and for a flat interface, simulations indicate that a coupling loss transition below ${0.5}\,\hbox {dB}$ and back reflection lower than ${-30}\,\hbox {dB}$ can be achieved, which is in line with design strategies for active-to-passive transitions^21,22^.

**Table 1:** Geometric parameters of the waveguide-to-waveguide interface. The values for the coupling loss (*CL*) and the back-reflection (*BR*) are recorded investigating the fundamental TE and TM cases. The back-reflection values are related in each case only to the fundamental mode. The cases of a flat and a tilted interface are presented.

$t_{core}$ $[\upmu \hbox {m}]$	$t_{slab}$ $[\upmu \hbox {m}]$	$l_{m}$ $[\hbox {nm}]$	$l_{SiO_{2}}$ $[\hbox {nm}]$	$\theta$ $[^\mathrm{o}]$	$l_{Wall,SiN}$ $[\hbox {nm}]$	$l_{Wall,SOI}$ $[\hbox {nm}]$	$w_{SiN}$ $[\upmu \hbox {m}]$	$w_{SOI}$ $[\upmu \hbox {m}]$	$t_{BOX}$ $[\upmu \hbox {m}]$	$t_{TOX}$ $[\upmu \hbox {m}]$	*CL* $[\hbox {dB}]$		*BR* $[\hbox {dB}]$	
											TE	TM	TE	TM
1.25	0.4	120	10	0	22	165	2.5	2.5	2	1	0.14	0.15	$-31.22$	$-32.28$
1.25	0.4	120	10	4	22	165	2.5	2.5	2	1	0.46	0.49	$-32.63$	$-32.28$

**Figure 2:**
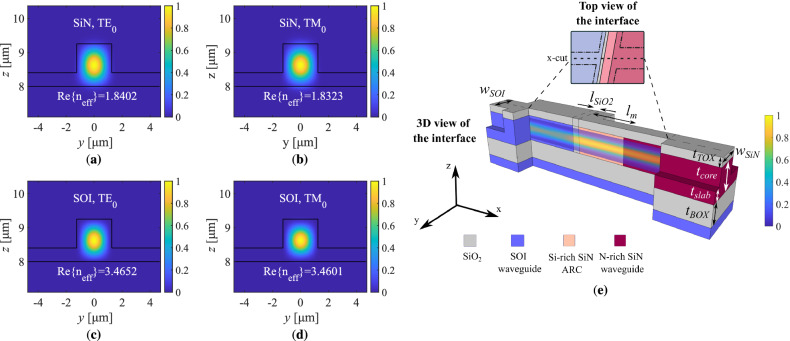
Calculation of the propagation characteristics regarding the fundamental TE and TM modes for the N-rich SiN and SOI waveguides. The distribution of the electric field at the cross-sections of the waveguides is calculated through the 2D-FDE method for the cases of **(a)** the $\hbox {TE}_{0}$ mode supported by the N-rich SiN waveguide, **(b)** the $\hbox {TM}_{0}$ mode supported by the N-rich SiN waveguide, **(c)** the $\hbox {TE}_{0}$ mode supported by the SOI waveguide and **(d)** the $\hbox {TM}_{0}$ mode supported by the SOI waveguide. The intensity of the electric field of the $\hbox {TE}_{0}$ mode through an x-cut at the centre of the waveguides is calculated through the 3D-FDTD method and is depicted along the 3D waveguide-to-waveguide interface in **(e)**, while the related geometric variables are noted. The intensity plot is depicted semi-transparently. The T-bar length variables and the top $\hbox {SiO}_{2}$ cladding at the areas of the slabs are not drawn for illustration purposes of the interface. The interface top view is provided as part of Fig. 1a, in order to depict the x-cut along the waveguides.

Through the commercial *MODE Solutions* package of Lumerical that implements the finite-difference (FDE) method, the modal distributions and the real parts of the effective indices of the fundamental TE and TM modes regarding the cross-sections of the two waveguides were numerically estimated and recorded in Fig. 2. The distributions refer to the intensity of the electric field for both modes. The intensity of the electric field of the propagating wave regarding the fundamental TE mode along the waveguide-to-waveguide transition through an x-cut at the center of the waveguide, is depicted as well.

The interconnection scheme was realised on an 8” SOI wafer with ${2}\,\upmu \hbox {m}$ thermally grown $\hbox {SiO}_{2}$ and ${1.25}\,\upmu \hbox {m}$ thick Si. Cavities were defined by ${248}\,\mathrm{nm}$ DUV lithography using a ${0.68}\,\upmu \hbox {m}$ thick M91Y resist and inductively coupled plasma (ICP) etching, down to a depth of ${1.25}\,\upmu \hbox {m}$ through an oxide hard mask. The non-etched areas were covered by PECVD $\hbox {SiO}_{2}$, while the Si-rich SiN and the N-rich SiN were deposited at 350 °C through the $\hbox {NH}_{3}$-free PECVD process described in^16^. Chemical mechanical polishing (CMP) was applied to planarize the N-rich SiN layer to the top level of the Si epilayer. After the planarisation step, the SiN and SOI waveguides were formed consecutively through DUV lithography using 1 and ${0.68}\,\upmu \hbox {m}$ M91Y resist, respectively, and ICP etching. The waveguides were finally capped by PECVD $\hbox {SiO}_{2}$, with a thickness of ${1}\,\upmu \hbox {m}$.

## Results and discussion

The spectral response of the devices was characterised using an Agilent 8164B tunable laser source with a wavelength tuning range between ${1260}\,$ and ${1360}\,\hbox {nm}$. The polarisation of the light was controlled to achieve either the TE or the TM excitation of the modes. Multiple cut-back structures with an increasing number of interfaces interconnected by N-rich SiN and SOI sections were defined to estimate the coupling loss and the back-reflection level with a different geometric parameter to be varied at each structure. Specifically, a parametric analysis was conducted (a) for the width of the interface and (b) the width of the SOI waveguide while keeping the N-rich SiN waveguide ${2.5}\,\upmu \hbox {m}$ wide. Three different chips located at the center of the wafer were characterised in order to take into account any statistical variation of the measurements.

**Figure 3:**
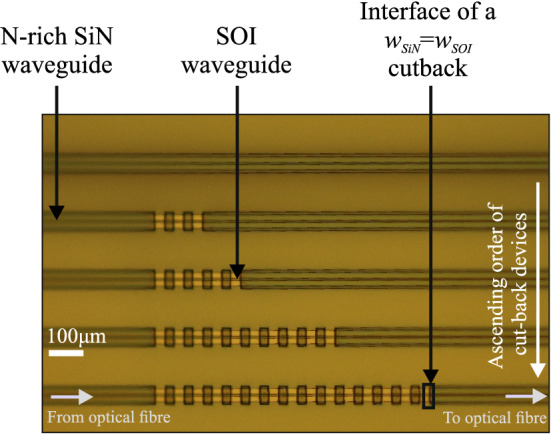
Microscope image of the cut-back structures used to measure the coupling loss between the N-rich SiN and the SOI waveguide for the case of varying interface width.

Figure 3 shows an image from an optical microscope of the fabricated devices for the cases of varying interface widths, that involve an increasing number of interfaces from 6 up to 30, starting from the top device down to the bottom one. The light is fed and collected by single-mode fibers through fiber grating couplers with straight gratings realised on the N-rich SiN waveguides. The width of the gratings is ${10}\,\upmu \hbox {m}$, their period is ${976}\,\hbox {nm}$, they have a filling factor of 50% and 30 teeth in total. A ${400}\,\upmu \hbox {m}$ long adiabatic taper narrows down the N-rich SiN waveguide from ${10}\,$ to ${1.1}\,\upmu \hbox {m}$, which is its single-mode width, allowing the adiabatic transition of the fundamental mode. A ${50}\,\upmu \hbox {m}$ long single-mode wide straight section follows, acting as a filter for the remaining high order modes that could appear during the excitation of the grating. Afterwards, a ${400}\,\upmu \hbox {m}$ long adiabatic taper is used for the transition of the single-mode width to the width of the interface, that varies from ${2.1}$ to ${3.1}\,\upmu \hbox {m}$, and consequently only the fundamental mode is guided to the interface. At each of the interfaces, the width of each of the waveguides allow the existence of high order modes that can be excited at each interface. These high order modes can propagate bidirectionally and independently along with the fundamental, through the recurrent interfaces connected with ${30}\,,\upmu \hbox {m}$ and around ${29,740}\,\upmu \hbox {m}$ long sections of SOI and N-rich SiN waveguides, respectively. Regarding their forward propagation, at the output of the recurrent interfaces the same scheme of the adiabatic tapers and the single-mode wide straight section as in the input is used. The filtering of any high order modes excited at the interfaces is therefore attained and the fundamental mode is guided adiabatically to the grating coupler. The single-mode wide straight section located at the input acts again as a filter for backward propagation caused by reflections at each of the interfaces. As a result, only the light and the corresponding information related to the fundamental mode reach the optical fiber at the output, while unwanted reflections from high order modes are also suppressed at the input taper.

The propagation losses related to the N-rich SiN and SOI waveguides have not been measured, as they are assumed to be negligible compared to the interface loss. A typical level of ${1}-{2}\,\hbox {dB/cm}$ for thin SiN^16^ and SOI^17^ waveguides, is an upper level for the thick waveguides in which there is less modal overlap with the waveguide side walls^23^. The total induced propagation loss is expected to be one order of magnitude lower than the loss of the interfaces. In particular, for the device of maximum 30 interfaces, the total propagation loss is $\sim$ ${0.2}\,\hbox {dB}$, while the total interface loss is $\sim$ ${4.5}\,\hbox {dB}$ based on the simulation data of Table 1. Nevertheless, it must be noted that the measured interface loss includes the loss associated to the length of both waveguide materials between the interfaces.

### Coupling loss

**Figure 4:**
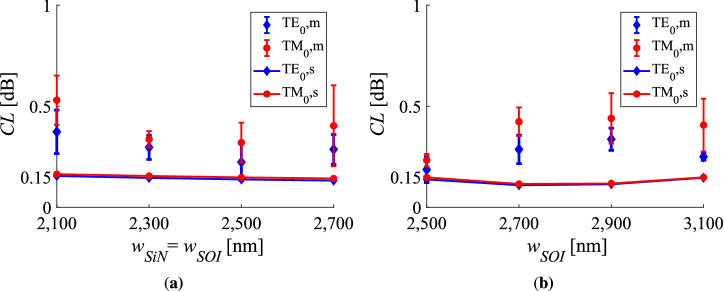
Characterisation data for the fundamental TE and TM modes, along with the simulated values at ${\uplambda }={1310}\,\hbox {nm}$. **(a)** Coupling loss under varying width of the interface, when the two waveguides have equal widths and **(b)** varying width of the SOI waveguide, when the width of the SiN waveguide is set at ${2.5}\,\upmu \hbox {m}$. The angle of the interface is set at $0^\circ$. The index *m* denotes measurement data, while *s* corresponds to simulation results. The characterisation data and the error bars refer to the mean value and the standard deviation of the cut-back losses, respectively.

In Fig. 4 the coupling loss derived from the cut-back measurements and the simulations regarding a flat interface, is depicted for different variations of the geometric parameters at the wavelength of ${1310}\,\hbox {nm}$. Figure 4a shows the coupling loss results when the interface width is varied from ${2.1}\,\upmu \hbox {m}$ up to ${2.7}\,\upmu \hbox {m}$. In Fig. 4b, characterisation and simulation data are plotted for a varying width of the SOI waveguide spanning from ${2.5}$ up to ${3.1}\,\upmu \hbox {m}$, while the corresponding width of the N-rich SiN waveguide is set at ${2.5}\,\upmu \hbox {m}$. The simulations in Fig. 4a reveal a decrease in coupling loss as the widths of both waveguides have the same increasing value, while a minimum is observed in Fig. 4b.

**Figure 5:**
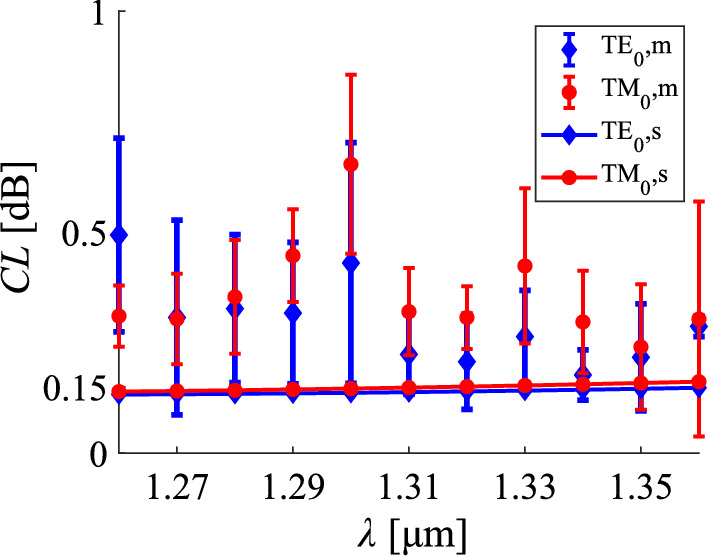
Characterisation data for the fundamental TE and TM modes, along with the simulated values. Coupling loss under varying wavelength along the O-band, when the widths of the two waveguides equal the ${2.5}\,\upmu \hbox {m}$ value and the angle is set at $0^\circ$. The index *m* denotes measurement data, while *s* corresponds to simulation results. The characterisation data and the error bars refer to the mean value and the standard deviation of the cut-back losses, respectively.

In Fig. 5, the coupling loss values for different wavelengths in the O-band are presented regarding the measured and the simulated data. The values refer to the specific case of equal widths being set at ${2.5}\,\upmu \hbox {m}$ and for a $0^\circ$ facet. The theoretical values show a coupling loss that is almost independent of the optical wavelength for the full O-band range.

The simulation curves in Figs. 4 and 5 show that the variations of the interface width, the SOI width and the wavelength affect minimally the coupling loss. This can be attributed to the large size of the waveguides under investigation. The coupling loss is the sum of the modal profile mismatch loss and the reflection-related loss caused by the modal effective refractive index difference. By investigating the interface width variation, a monotonic decrease of the coupling loss is expected as the interface width is increased. For small widths the modes of the waveguides interact more with the $\hbox {SiO}_{2}$ cladding, causing a decrease on the effective refractive indices. On the one hand, that decrease violates the DLARC condition, increasing the back-reflection and consequently the related loss. Furthermore, given the different confinement factors of the two waveguides (lower in SiN than in SOI) the interface width decrease introduces a discrepancy between the modal profiles enhancing the mode mismatch. The coupling loss is expected to converge as the waveguide width is increased, given the alleviation of the modal profile discrepancy and the convengence of the effective refractive indices. A similar explanation can be given for the SOI width variation, however the coupling loss is expected to increase more rapidly as the SOI width is decreased. In addition to the modal profile discrepancy and the anti-reflection condition violation, the actual mode diameter difference deteriorates further the coupling performance. It is worth noting that a minimum of the coupling loss is expected at an optimal SOI width value. In Fig. 2b that minimum lies around the ${2.7}\,\upmu \hbox {m}$ width. Nevertheless, for fabrication complexity reasons the nominal width of ${2.5}\,\upmu \hbox {m}$ was chosen, which results in almost the same coupling loss level. Because of the limited design space layout, only devices of large widths have been taken into account targeting a low coupling loss behavior. As a result, the low and almost unaffected coupling loss values for varying widths stems from the restricted range of large widths used in the experiments. As for the wavelength variation, the introduced modal dispersion affects the modal effective refractive index difference and the modal profile overlap, while the wavelength-dependent anti-reflection condition is not satisfied for non-nominal wavelengths. However, the small perturbations on the modal characteristics in the restricted O-band range do not increase significantly the coupling loss level.

### Back-reflection

**Figure 6:**
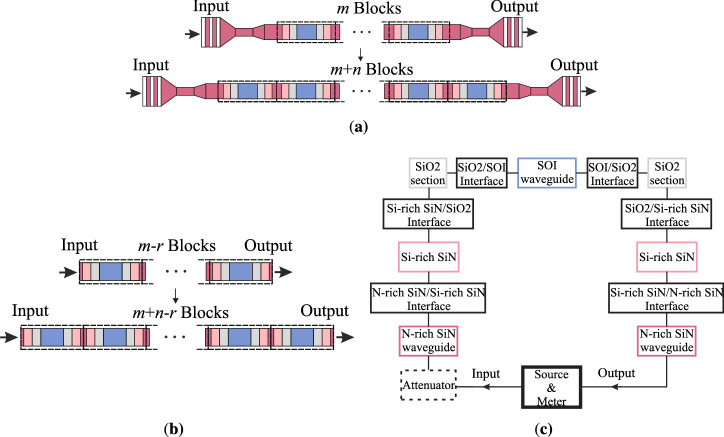
Top view of the cut-back devices **(a)** in their designed form including the I/O parts and **(b)** after their normalisation with a device of reference that contains *r* blocks. Each device is composed of a number of repeated blocks, that after normalisation is reduced by *r*. The block appears enclosed in the dashed frame and is composed of two N-rich SiN, two Si-rich SiN, two $\hbox {SiO}_{2}$ and one SOI sections. In **(c)** the block diagram used for the modelling of one block is depicted.

The cut-back structures for the coupling loss characterisation can be used for the estimation of the back-reflection that each interface introduce. Towards this aim, the spectral response of the devices of the cut-back sets is simulated and is fitted to the measured data, having as independent variables the insertion and the return loss of each interface, as well as its geometrical characteristics. Their calculation is based on the concept that is depicted in Fig. 6. As can be seen in Fig. 6a, along with the fiber-to-chip I/O parts, each device contains a number of repeated blocks. The block is the region enclosed in the dashed frame and is composed of two N-rich SiN, two Si-rich SiN, two $\hbox {SiO}_{2}$ and one SOI section. The normalisation of the transmission of the devices with the transmission of a reference device, removes the information related to the I/O parts as shown in Fig. 6b. As a result, the characteristics of the spectra of the normalised devices can be attributed to the blocks they contain.

Through the commercial *INTERCONNECT* package of Lumerical and by implementing the scattering matrix method, transmission models for the normalised devices are set up. The block diagram of the model for one block is presented in Fig. 6c. The model has been set up using the primary elements that *INTERCONNECT* provides. In specific, for the N-rich SiN and SOI sections the bidirectional straight optical waveguide element was used, that takes as inputs the modal optical propagation characteristics and the length of the waveguide. For the N-rich SiN/Si-rich SiN/$\hbox {SiO}_{2}$/SOI interface, the bidirectional optical mirror and the bidirectional straight optical waveguide elements were used. The reflectivity and the transmissivity of an incident signal at the N-rich SiN/Si-rich SiN, Si-rich SiN/$\hbox {SiO}_{2}$ and $\hbox {SiO}_{2}$/SOI interfaces were defined through the optical mirror element, while the waveguide element models the Si-rich SiN and $\hbox {SiO}_{2}$ sections between the interfaces. In addition, in order to take into account the interface loss that is not related to the reflectivity, an attenuator was used for the adjustment of the loss level. The same concept is followed for devices that contain more blocks, by cascading multiple times the model that refers to one block. The normalisation device contains three blocks and the maximum number of blocks in a device are fifteen. The optical and the geometrical parameters used for the simulation are summarised in Table 2. The fitting of the simulated and the measured response is done in the frequency domain. The calculation of the attenuation $A_{1}$ and the reflectivities $R_{1},R_{2},R_{3}$ associated with each interface, is done for devices that contain seven blocks and for three different chips located at the center of the wafer in order to include any statistical variations. It is noted that the fitted values compose a set capable of replicating the measured response, however this is not unique for a 6-variable problem and therefore the calculated values provide a reasonable assumption of the order of magnitude of the related variables.

The back-reflection from the interfaces leads to destructive interference at specific wavelengths creating dips at the spectrum. The position and the depth of the dips are related to the optical length of the multiple cavities of the devices, along with the reflectivity of the interfaces and the total attenuation. The length of the SOI waveguide is constant based on the design layout, on the contrary to the lengths of the N-rich SiN, Si-rich SiN and $\hbox {SiO}_{2}$ sections that are varying. In specific, the lengths of the Si-rich SiN and $\hbox {SiO}_{2}$ sections depend on the deposition, and in that way they define the length of the N-rich SiN waveguide by changing the length of the opened cavity. The free spectral ranges (FSR) of the spectra were matched through the fitting of the effective index of the SOI waveguide, while all the other effective and group indices of the waveguides are based on simulations. The level of the attenuation defines the minimum insertion loss of the device. The reflectivity and the transmissivity of the N-rich SiN/Si-rich SiN, Si-rich SiN/$\hbox {SiO}_{2}$ and $\hbox {SiO}_{2}$/SOI interfaces are assumed to be constant in the O-band. The measured normalised transmission used for the fit is not ideally flat in the O-band, due to a non-perfect offset between the spectra of the device under investigation and the device of reference. In order to fit the measured and the simulated data, a restricted range of wavelengths was chosen where the normalised response appears flat. However, a fit for each of the dips can be achieved given a local adjustment of the attenuator $A_{1}$. A device composed of seven blocks was chosen for the fitting, where deeper resonances are identified clearly and dominate the transmission response compared to other sources of reflection, such as sidewall roughness. The total reflectivity *R* of the N-rich SiN/Si-rich SiN/$\hbox {SiO}_{2}$/SOI interface is calculated through a model including the three optical mirrors of $R_{1}$, $R_{2}$ and $R_{3}$ values, respectively, along with the Si-rich SiN and the $\hbox {SiO}_{2}$ sections between them. The total attenuation *A* is the sum of the attenuation $A_{1}$ and the loss related to the reflectivity of the interface. The calculated values refer to the reflectivity and the attenuation at ${1310}\,\hbox {nm}$.

**Table 2:** Simulation parameters for the definition of the theoretical model of one block. The reflectivities $R_{1}$, $R_{2}$, $R_{3}$, *R* of the interfaces and the attenuations $A_{1}$, *A* are calculated through the fitting of the simulated and the measured spectra.

N-rich SiN waveguide	Si-rich SiN waveguide	$\hbox {SiO}_{2}$ section	SOI waveguide	Interfaces	Attenuators
$n_{eff,TE}$=1.8402 (sim)	$n_{eff,TE}$=2.4865 (sim)	$n_{eff,TE}$=1.4328 (sim)	$n_{eff,TE}$=3.3609 $\pm 0.09$ (fit)		
$n_{g,TE}$=1.9382 (sim)	$n_{g,TE}$=2.5825 (sim)	$n_{g,TE}$=1.4744 (sim)	$n_{g,TE}$=3.6797 (sim)	$R_{1}$ (fit)	
$n_{eff,TM}$=1.8323 (sim)	$n_{eff,TM}$=2.4790 (sim)	$n_{eff,TM}$=1.4451 (sim)	$n_{eff,TM}$=3.4515 $\pm 0.06$ (fit)	$R_{2}$ (fit)	$A_{1}$ (fit)
$n_{g,TM}$=1.9495 (sim)	$n_{g,TM}$=2.5956 (sim)	$n_{g,TM}$=1.4618 (sim)	$n_{g,TM}$=3.6896 (sim)	$R_{3}$ (fit)	*A* (fit)
$l=14.871$ $\pm 0.17$ $\,\upmu \hbox {m}$ (fit)	*l*=128.75 $\pm 17.15$ $\,\hbox {nm}$ (fit)	*l*=21.5 $\pm 13.26$ $\,\hbox {nm}$ (fit)	*l*=${30}\,\upmu \hbox {m}$ (design)	*R* (fit)	
$loss=0$ dB/m	$loss=0$ dB/m	$loss=0$ dB/m	$loss=0$ dB/m		

**Figure 7:**
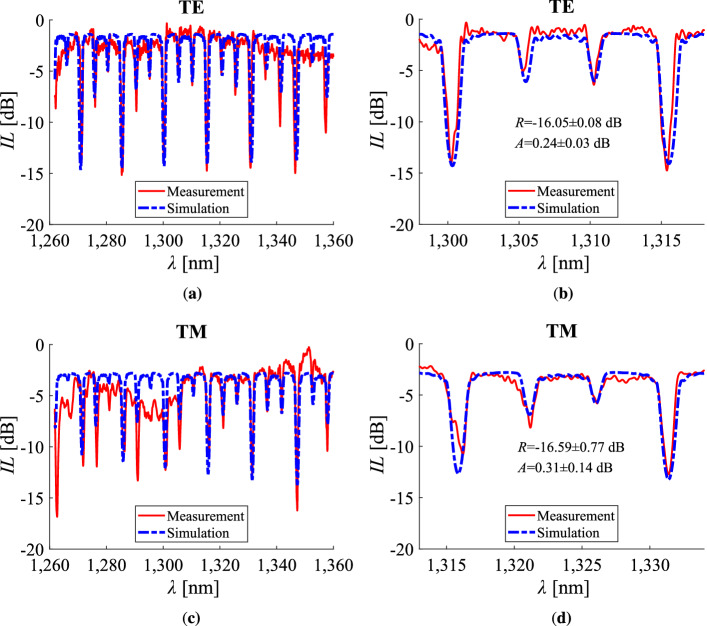
Transmission spectrum for a normalised device of seven blocks, **(a)**, **(c)** in the O-band and **(b)**, **(d)** in a restricted range of wavelengths, for TE and TM polarisations, respectively. The geometrical parameters of the device are summarised in Tables 1 and 2.

In a mathematical framework, the fit is performed between the simulated and the experimental values of the $20 \, \log |S_{21}|$ element of the total S-matrix of the two-port device. The calculation of the total S-matrix is done by the S-parameter Simulator (SPS) solver of *INTERCONNECT*^24^. The fit of the spectra along with the calculated reflectivity of the N-rich/Si-rich SiN/$\hbox {SiO}_{2}$/SOI interface and the total attenuation for both TE and TM polarisations is presented in Fig. 7. The geometrical parameters of the device are summarised in Table 1 regarding a ${0^\circ }$ interface, and in Table 2. The response related to the device of one of the three chips is presented for both TE and TM polarisations. In Fig. 7a and c the measured and the simulated response is plotted across the O-band, while in Fig. 7b and d the restricted range of wavelengths that was used for the the fitting is provided. For each polarisation and based on the characterisation results of the three different chips, the reflectivity ${1310}\,\hbox {nm}$ is noted in a range defined by the standard deviation of the data. In specific, this is ${-16.05 \pm 0.08}\,\hbox {dB}$ and ${-16.59 \pm 0.77}\,\hbox {dB}$ for the TE and TM cases, respectively. The calculated losses of ${0.24\pm 0.03}\,\hbox {dB}$ for TE and ${0.31 \pm 0.14}\,\hbox {dB}$ for TM at the central wavelength of ${1310}\,\hbox {nm}$ is in line with the results presented in Fig. 5, acting as an indication for the quality of the fit.

## Discussion

### Statistical analysis

Through Figs. 4, 5 and 7, an indication for a sub-dB coupling loss and ${-16}\,\hbox {dB}$ back-reflection transition is revealed. The discrepancy between the calculated and the simulated values could be attributed to the potential variations of the geometrical parameters and the material properties of the related layers that compose the interfaces. In order to evaluate the deterioration of the interface quality due to the fabrication errors, the transition metrics were theoretically calculated under the random variation of the simulation inputs. Specifically, uniform random numbers in a restricted range were given to the variables that refer to the geometrical and material parameters that can vary during the fabrication. The geometrical parameters include the lengths of the T-bars $l_{Wall,SiN}$ and $l_{Wall,Si}$, the thicknesses of the waveguide cores $t_{core,Si}$ and $t_{core,SiN}$, the thicknesses of the waveguide slabs $t_{slab,Si}$ and $t_{slab,SiN}$, the lengths of the matching layers $l_{m}$ and $l_{\hbox {SiO}_{2}}$ and the lateral misalignment of the waveguides $y_{mis}$. Concerning the material properties, the refractive indices ${n_{N-rich SiN}}$ and ${n_{Si-rich SiN}}$ of the involved silicon nitride layers were investigated. The variation of the geometrical variables is ${\pm 20}\,\hbox {nm}$ from the nominal value, while the corresponding variation for the refractive indices is ${\pm 0.02}$. All the other parameters retain their nominal values, as described in Table 1 for the flat and ${2.5}\,\upmu \hbox {m}$ wide interface case.

**Figure 8:**
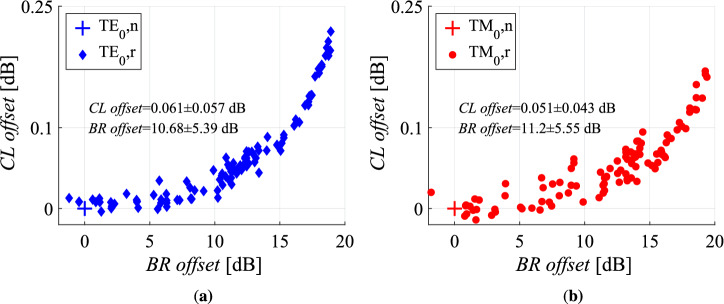
Scatter diagrams for the simulated offset of the coupling loss and the back-reflection metrics, when uniform random numbers in a restricted range are set to the varying geometrical and material parameters that define the quality of the transition. A variation of ${\pm 20}\,\hbox {nm}$ from the nominal value is assumed for the geometrical variables and a change of ${\pm 0.02}$ is accounted for the refractive indices of the materials. Nominal values are set to all the other parameters, as described in Table 1 for the flat interface case. Both TE and TM polarisations are studied. The wavelength is set at ${1310}\,\hbox {nm}$. The indices *n* and *r* refer to the nominal and the random cases, respectively.

In Fig. 8 scatter plots regarding the behavior of the interface under the multi-variable variations are presented. In particular, the offset of the coupling loss and the back-reflection from the nominal value is plotted for all the different uniform random sets of values. The crosses represent the nominal values, while the diamonds and the circles correspond to the randomly calculated metrics. The number of the sets is 100, while the study concerns the fundamental modes of both TE and TM polarisations. In addition, the mean value of the offsets along with their standard variation are noted on the graphs. The variation of the values induce a degradation of the interface quality, as both the coupling loss and the back-reflection mostly increase.

It can be seen, that the back-reflection metric is affected more than the coupling loss. High reflectivity values can be attributed mostly to the violation of the anti-reflective mechanism through the variation of the lengths of the matching layers and the refractive indices of the silicon nitride sections. For those values, the coupling loss is defined mainly by the level of the reflectivity and less by the geometrical discrepancies between the waveguides. The sum of the offset and the simulation data of Table 1 results in ${-20.52 \pm 5.39}\,\hbox {dB}$ (${-21.08 \pm 5.55}\,\hbox {dB}$) for the TE (TM) mode, which overlaps with the measurement data for the reflectivity that are ${-16.05 \pm 0.08}\,\hbox {dB}$ (${-16.59 \pm 0.77}\,\hbox {dB}$) for the TE (TM) mode. It is noted, that this high value of the reflectivity implies that an accurate realisation of the geometrical and the optical characteristics of the DLARC is necessary.

The discrepancy observed between the measured and the simulated coupling loss values, can be attributed to the offset presented in Fig. 8. By adding the offset to the simulation data of Table 1, the loss of ${0.201 \pm 0.057}\,\hbox {dB}$ (${0.201 \pm 0.043}\,\hbox {dB}$) for the TE (TM) mode is attained. This range overlaps with the values for a ${2,500}\,\hbox {nm}$ wide interface in Fig. 4a and b, which are ${0.22 \pm 0.091}\,\hbox {dB}$ (${0.32 \pm 0.098}\,\hbox {dB}$) and ${0.19 \pm 0.064}\,\hbox {dB}$ (${0.23 \pm 0.031}\,\hbox {dB}$) for the TE (TM) mode.

### Performance comparison

**Table 3:** Integration schemes in literature between SiN waveguides and thick devices compared to the scheme under investigation of the underlying work. The SiN waveguides noted refer to the related single-mode sections. The thick III-V buffers are not taken into account for the total thickness of the III-V stacks.

Reference	Coupling scheme	*CL* (dB)		*BR* (dB)		Band	SiN Platform *W* x *H* (${\upmu \hbox {m}^2}$)	Thick Device *W* x *H* (${\upmu \hbox {m}^2}$)
		TE	TM	TE	TM			
This work	Butt-coupling	$<0.5$	$<0.5$	$-16.05 \pm 0.08$ (${1310}\,\hbox {nm}$)	$-16.59 \pm 0.77$ (${1310}\,\hbox {nm}$)	O	N-rich SiN 1.1 × 1.25	SOI 2.5 × 1.25
^25^	Butt-coupling + Bi-layer Coupling	$>9$ (sim)	–	–	–	C	$\hbox {Si}_{3}\hbox {N}_{4}$ 1 × 0.4	InGaAsP MQW stack 4 × 2.378
^26^	Butt-coupling + Bi-layer Coupling	$>7.63$ (sim)	–	–	–	C	$\hbox {Si}_{3}\hbox {N}_{4}$ 1 × 0.4	InGaAsP MQW stack 5 × 3
^27^	Butt-coupling + Evanescent Coupling	$<1$ (sim)	–	–	–	O + C	$\hbox {Si}_{3}\hbox {N}_{4}$ 0.6 x 0.6	Ge 0.6 × 0.8
^28^	Butt-coupling + Evanescent Coupling	1.03 (sim)	–	− 52.6 (sim)	–	C	$\hbox {Si}_{3}\hbox {N}_{4}$ 0.8 × 0.8	InP QD stack 2 × 3
^29^	Butt-coupling + Evanescent Coupling	1.2 (sim)	–	–	–	C	$\hbox {Si}_{3}\hbox {N}_{4}$ 1 × 1	Ge 0.59 × 0.8
^30^	Butt-coupling + Evanescent Coupling	0.95 (sim)	–	−30 (sim)	–	C	$\hbox {Si}_{3}\hbox {N}_{4}$ 0.8 × 0.8	InP QD stack 2 × 3
^31^	Butt-coupling	1.25 (sim)	–	–	–	O	$\hbox {Si}_{3}\hbox {N}_{4}$ 1 × 1	Ge/SiGe MQW stack 1 × 1.3

Table 3 compares the coupling performance of the underlying interconnection scheme with other works available in the literature that are related to the integration of SiN waveguides to thick devices. We record, if available, the coupling loss and the back-reflection values for the fundamental TE and TM modes, the coupling technique that was used, the operational frequency range and the characteristics of the thick device in terms of its incorporated material and the dimensions of its cross-section. In order to provide an order of size, a comparison of the volume of both the SiN waveguide and the corresponding thick device is shown in the two columns in the right hand side of the table. All of the related works identified in the literature focus on results from simulations. We observe that the demonstrated performance of this work in terms of the coupling loss regarding TE polarisation is improved by a factor of 2 compared to other published simulated work. No data was found in the literature for TM polarisation which set our structure as the leading demonstrator for this particular architecture.

### SiN-to-MQW interconnection

**Figure 9:**
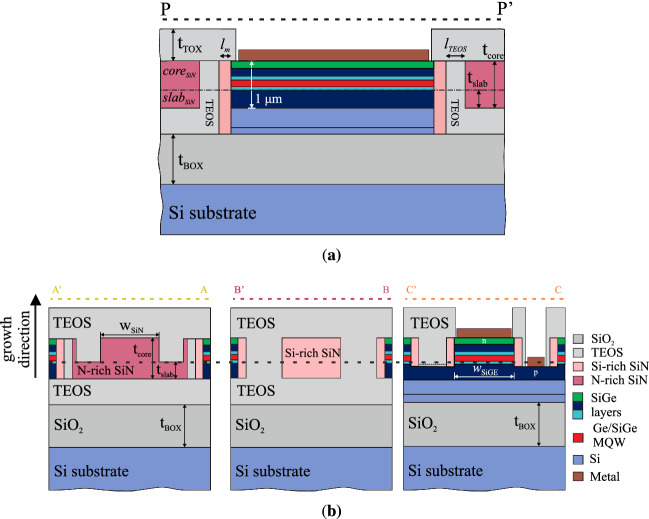
SiN-to-MQW butt-coupling interconnection concept. The PP’ dashed line reveals the side view of the structure depicted in **(a)**. The MQW stack stands in the middle and its facets are connected to the N-rich SiN waveguides. Cross-sections of the separate elements of the structure are disclosed in **(b)**. The cuts AA’ and CC’ reflect to the N-rich SiN and the MQW SiGe-stack, respectively. BB’ refers to the Si-rich SiN layer that is set, along with the TEOS layer, between the two waveguides towards optimal photonic coupling. All the materials are described in the colormap of **(b)**.

The aforementioned results and the analysis set the basis for a structure providing a SiN-to-MQW waveguide interconnection. The SiN-to-MQW scheme is depicted in Fig. 9, where the MQW region is a generic SiGe-based stack of Ge wells. The stack is a top-down p-i-n diode with multiple QWs in the intrinsic region. The total thickness of the stack is ${1}\,\upmu \hbox {m}$. The effective index of the supported $\hbox {TE}_{0}$ mode for a ${2.5}\,\upmu \hbox {m}$ wide SiGe waveguide is 4.0133. The layers of the stack are depicted in the material colormap in Fig. 9b. The lateral-view in Fig. 9(b) shows a straight MQW waveguide connected from both sides to N-rich SiN waveguides (*n* $=$ 1.9). At their interface, tetraethyl orthosilicate (TEOS) (*n* $=$ 1.45) and Si-rich SiN (*n* $=$ 2.76) layers are placed. The TEOS/Si-rich ARC scheme is used similarly to the SOI-SiN case. The dashed lines AA’, BB’ and CC’ define the cross-sections of the N-rich SiN waveguide, the Si-rich SiN layer and the SiGe stack, respectively, as shown in Fig. 9a.

Following the same procedure as in the SiN-to-SOI interconnection, the optimal geometric parameters were identified through FDE and 3D-FDTD simulations and are presented in Table 4, along with the resulting values for the coupling loss and the back-reflection for the fundamental TE mode. The results refer to the wavelength of ${1310}\,\hbox {nm}$ for a flat interface. Based on the simulations, a transition of coupling loss below ${0.5}\,\hbox {dB}$ and back reflection lower than ${-50}\,\hbox {dB}$ can be achieved.

**Table 4:** Geometric parameters of the SiN-to-MQW interface. The values for the coupling loss and the back-reflection at the wavelength of ${1310}\,\hbox {nm}$ are recorded investigating the fundamental TE case. The back-reflection value is related only to the fundamental mode.

$t_{core}$ $[\,\upmu \hbox {m}]$	$t_{slab}$ $[\,\upmu \hbox {m}]$	$l_{m}$ $[\hbox {nm}]$	$l_{TEOS}$ $[\hbox {nm}]$	$\theta$ $[^\mathrm{o}]$	$w_{SiN}$ $[\,\upmu \hbox {m}]$	$w_{SiGe}$ $[\,\upmu \hbox {m}]$	$t_{BOX}$ $[\,\upmu \hbox {m}]$	$t_{TOX}$ $[\,\upmu \hbox {m}]$	*CL* $[\hbox {dB}]$	*BR* $[\hbox {dB}]$
									TE	TE
1	0.4	122	241	0	2.5	2.5	2	1	0.34	$-50.37$

To further refine the transition data discussed on the ${1.25}\,\upmu \hbox {m}$-thick SiN-to-SOI demonstration and to align with the waveguide thickness proposed for the QCSE to SiN transition, two further fabrication runs related to a ${1}\,\upmu \hbox {m}$ thick SiN-to-SOI waveguides were conducted. The results of all three runs at the wavelength of ${1310}\,\hbox {nm}$ are summarised in Table 5. The recorded values demonstrate consistently a ${0.5}\,\hbox {dB}$ coupling loss value and a better than −16dB back-reflection value for all waveguide thicknesses and different fabrication runs. Consequently, the proposed fabrication process shows promising results and robustness for a potential ${1}\,\upmu \hbox {m}$-thick SiN-to-MQW integration.

**Table 5:** Coupling loss and back-reflection values for three fabrication runs involving ${1.25}\,\upmu \hbox {m}$ and ${1}\,\upmu \hbox {m}$ -thick SiN and SOI waveguides. Both TE and TM cases are presented. The back-reflection values are related in each case only to the fundamental mode.

Run	$t_{core}$ $[\,\upmu \hbox {m}]$	$t_{slab}$ $[\,\upmu \hbox {m}]$	*CL* $[\hbox {dB}]$		*BR* $[\hbox {dB}]$	
			TE	TM	TE	TM
1	1.25	0.4	${0.22 \pm 0.09}$	${0.32 \pm 0.10}$	${-16.05 \pm 0.08}$	${-16.59 \pm 0.77}$
2	1	0.4	0.47	0.49	${-17.73}$	${-16.10}$
3	1	0.4	0.5	0.58	${-18.77}$	${-19.56}$

## Conclusions

We have demonstrated an integrated butt-coupling scheme between thick N-rich SiN and SOI rib waveguides for operation in the O-band with ${<0.5}\,\hbox {dB}$ coupling loss and better than ${-16}\,\hbox {dB}$ back-reflection at ${1310}\,\hbox {nm}$ for both TE and TM polarisations. To the best of our knowledge, this is the first demonstration of such an integration concept with transmission and reflection data reported for TE and TM polarisations. The tunability of silicon nitride, along with its BEOL integration capability, render it as a valid solution for the interconnection of multi-layer micro-metric stacks and offers a path to simple evanescent coupling with a compact SOI platform. Based on the demonstrated results, a potential integration between an N-rich SiN waveguide and a SiGe-based MQW stack has also been discussed, setting the ground for a process and temperature tolerant interconnection concept between thick active devices grown on SOI and an interface layer based on SiN. The proposed micro-metric active-to-passive integration scheme is expected to offer a CMOS based multi-material integration pathway for next-generation data centre optical transceivers, exploiting the temperature tolerant low-loss routing and bridging-to-SOI capabilities of SiN, in combination with the fast and low power consuming modulation performance of compact MQW structures.

## Data Availability

The datasets used and analysed during the current study are available from the corresponding author on reasonable request.
